# Antifungal Effect and Inhibition of the Virulence Mechanism of D-Limonene against *Candida parapsilosis*

**DOI:** 10.3390/molecules27248884

**Published:** 2022-12-14

**Authors:** Melyna Chaves Leite-Andrade, Luiz Nascimento de Araújo Neto, Maria Daniela Silva Buonafina-Paz, Franz de Assis Graciano dos Santos, Adryelle Idalina da Silva Alves, Maria Carolina Accioly Brelaz de Castro, Edna Mori, Bruna Caroline Gonçalves Vasconcelos de Lacerda, Isaac Moura Araújo, Henrique Douglas Melo Coutinho, Grażyna Kowalska, Radosław Kowalski, Tomasz Baj, Rejane Pereira Neves

**Affiliations:** 1 Departamento de Medicina Tropical, Universidade Federal de Pernambuco (UFPE), Recife 50670-901, PE, Brazil; 2 Departamento de Micologia, Universidade Federal de Pernambuco (UFPE), Recife 50670-901, PE, Brazil; 3 Laboratório de Parasitologia e Laboratório de Imunologia IAM, Centro Acadêmico de Vitória, Universidade Federal de Pernambuco (UFPE), Vitória de Santo Antão 55608-680, PE, Brazil; 4 Faculdade CECAPE College, São José, Juazeiro do Norte 63024-015, CE, Brazil; 5 Departamento de Química Biológica, Universidade Regional do Cariri, Crato 63105-010, CE, Brazil; 6 Department of Tourism and Recreation, University of Life Sciences in Lublin, 15 Akademicka Str., 20-950 Lublin, Poland; 7 Department of Analysis and Food Quality Assessment, University of Life Sciences in Lublin, 8 Skromna Str., 20-704 Lublin, Poland; 8 Department of Pharmacognosy with Medicinal Plants Garden, Medical University of Lublin, 1 Chodzki Str., 20-093 Lublin, Poland

**Keywords:** adherence, *Candida parapsilosis*, mechanism of action, morphogenesis, terpenoid

## Abstract

Yeasts from the *Candida parapsilosis* complex are clinically relevant due to their high virulence and pathogenicity potential, such as adherence to epithelial cells and emission of filamentous structures, as well as their low susceptibility to antifungals. D-limonene, a natural compound, emerges as a promising alternative with previously described antibacterial, antiparasitic, and antifungal activity; however, its mechanisms of action and antivirulence activity against *C. parapsilosis* complex species have not been elucidated. Therefore, in the present study, we aimed to evaluate the antifungal and antivirulence action, as well as the mechanism of action of D-limonene against isolates from this complex. D-limonene exhibited relevant antifungal activity against *C. parapsilosis* complex yeasts, as well as excellent antivirulence activity by inhibiting yeast morphogenesis and adherence to the human epithelium. Furthermore, the apoptotic mechanism induced by this compound, which is not induced by oxidative stress, represents an important target for the development of new antifungal drugs.

## 1. Introduction

The increased incidence of invasive yeast infections by the *Candida parapsilosis* complex is of great clinical relevance since its frequency as an invasive candidiasis agent has exceeded the isolation of *C. albicans* [1]. In addition, yeasts belonging to this complex have demonstrated a high virulence and pathogenicity potential, such as adherence to epithelial cells and emission of filamentous structures. These represent indispensable virulence factors for the establishment of the disease, given that they are responsible for the early stage of infection and invasion into a human host [2].

Moreover, although *C. parapsilosis* infections have generally resulted in lower morbidity and mortality rates as compared with *C. albicans* infections, certain authors have reported that several clinical isolates from these species were less susceptible to echinocandins, and in some regions, resistance to azole treatment has also been observed, which makes choosing the appropriate antifungal therapy difficult [3,4,5].

In addition, the available therapeutic options are limited and have led to increased mortality rates by *C. parapsilosis* complex species, with the search for new antifungal agents being evermore required [6]. Thus, natural plant-derived products, especially essential oils, have appeared as a therapeutic alternative, being described as promising treatments against pathogenic microorganisms [7,8].

Limonene is one of the main constituents of citrus oils and is the most widely used terpene in the food and beverage industry due to its pleasant fragrance and nontoxicity [6]. Limonene has also been shown to be an excellent microorganism population reducer, especially fungi. This compound has presented notable pharmacological activities in several studies, such as antimicrobial activity (in particular, antifungal activity) [9], antitumor activity [10], ascaricidal and insecticidal activity [11], and antiparasitic activity [12,13].

The mechanism of action of limonene against *Candida* yeasts, however, has not yet been fully understood. Thakre et al. [6] suggested that limonene induced cell wall and cell membrane damage leading to oxidative stress, brought about DNA damage, and induced apoptosis in *Candida albicans*. Still, information regarding its antifungal potential against *C. parapsilosis* complex yeasts, its mechanism of action, as well as its activity in controlling virulence factors produced by these microorganisms is still scarce.

It is important, therefore, to elucidate the antivirulence action of limonene against *C. parapsilosis* complex yeasts, since inhibition of yeast adhesion and morphogenesis may prevent the establishment of infection, and thus, improve the prognosis of patients with invasive candidiasis. Given the above, in the present study, we aimed to evaluate the antifungal action, antivirulence, and mechanism of action of D-limonene against clinical isolates from the *C. parapsilosis* complex.

## 2. Results

### 2.1. Antifungal Activity

The D-limonene antifungal activity evaluation against the used *C. parapsilosis* complex strains presented significant results, the MIC values being 256 µg/mL, 512 µg/mL, and 1024 µg/mL, with fungicidal activity. However, 100% cell inhibition was not observed in some isolates, these presenting MIC values ≥ 1024 µg/mL (Table 1).

The commercial antifungals fluconazole, amphotericin B, micafungin, anidulafungin, and caspofungin were employed as control drugs. All strains used in the study were sensitive to fluconazole and amphotericin B, with MIC values ranging from 0.125 to 1 µg/mL for echinocandins, while the MIC values ranged from 0.06 to 4 µg/mL for caspofungin, with the isolates obtaining MIC values of 4 µg/mL being considered to be dose-dependent isolates. Micafungin varied from 0.03 to 4 µg/mL, and also presented dose-dependent isolates (MIC 4 µg/mL); anidulafungin, in contrast, varied from 0.03 to 8 µg/mL, presenting dose-dependent (MIC 4 µg/mL) and resistant (≥8 µg/mL) isolates.

In this study, echinocandin resistant and dose-dependent isolates were observed. 

### 2.2. Morphogenesis Assay

**Figure 1:**
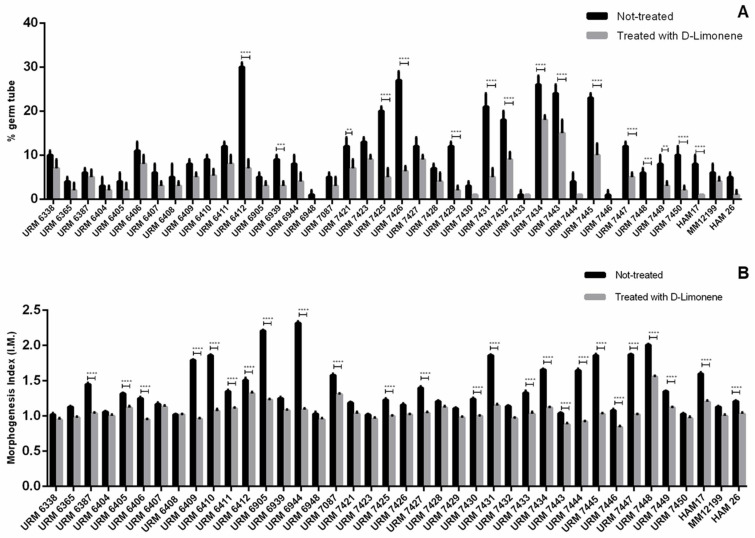
Evaluation of yeasts morphogenesis inhibition of *Candida parapsilosis* complex by D-limonene: (**A**) Germ tube emission after 1 h of incubation; (**B**) morphogenesis index (IM) after 3 h of incubation. Data represent mean and standard deviation in triplicate (*n* = 3). For the analysis, a Tukey′s multiple comparison test was performed for all means obtained at a significance level of 5%. The symbols “**”, “***”, “****” indicate significant differences between germ tube emission percentage and morphogenesis index (*p* ≤ 0.001), (*p* ≤ 0.0003), and (*p* ≤ 0.0001), respectively.

Germination tube production (lateral evagination) was evaluated with the 40 isolates after 1 h of incubation. The percentage of cells emitting germination tubes ranged from 2 to 30%, and this production was not specific to each species, but inherent to each evaluated strain. However, when evaluated in the presence of D-limonene, a decrease in the production of this virulence factor was found, ranging from complete germination tube formation inhibition, up to 18% (Figure 1A).

The index of morphology (I.M) was determined by analyzing the cells incubated for 3 h, with an average I.M of 1.32 being observed for strains from the *C. parapsilosis* complex, indicating a prevalence of blastoconidia forms. Only the isolates URM6905, URM6944, and URM7448 presented cells predominantly in the pseudomycelium form, with IMs of 2.21, 2.32, and 2.01, respectively; however, the ability of these isolates to form true hyphae was not observed. This index was also reduced in the presence of D-limonene, which presented a maximum I.M value of 1.56 (Figure 1B).

### 2.3. Adherence Capacity Assay of Yeast Cells to Epithelial Cells

Three levels of epithelial cell adherence for species from the *C. parapsilosis* complex were observed. Among the 40 strains evaluated, 10 (22.5%) isolates stood out with strong adherence capacity, 25 (62.5%) isolates showed poor adhesion capacity, and five (12.5%) isolates showed no adhesion capacity to human epithelial cells.

**Figure 2:**
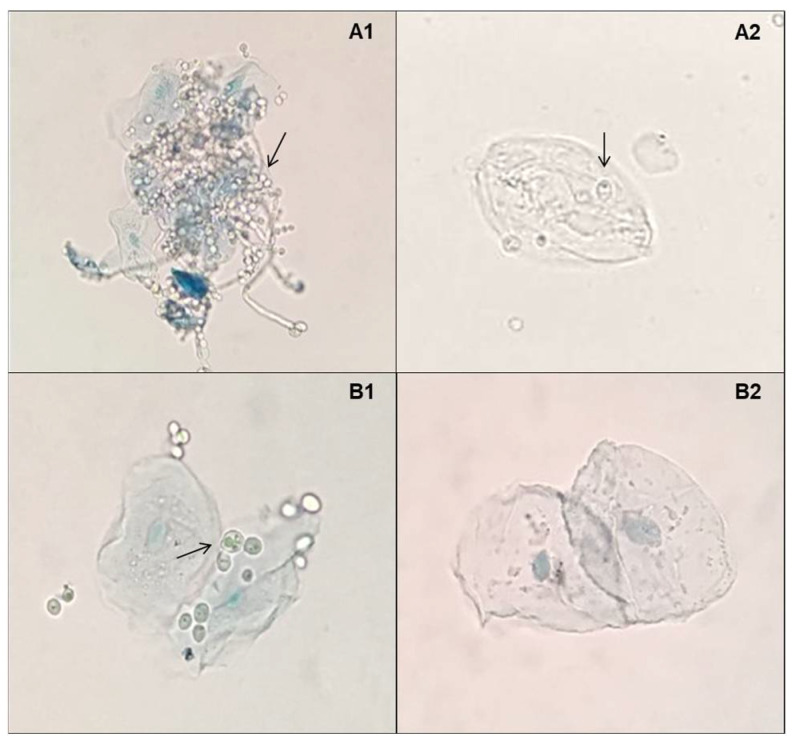
Inhibition of the ability to adherence to *Candida parapsilosis* complex yeast epithelial cells by D-limonene: (**A1**,**A2**) URM 7445 *Candida parapsilosis* stricto sensu showed strong adherence before treatment and poor adherence after treatment with D-limonene; (**B1**,**B2**) URM 7434 *Candida orthopsilosis* revealed poor adherence before treatment and absent adherence after treatment with D-limonene. Arrows indicate yeast cells adhered to human epithelial cells.

Yeast adhesion to epithelial cells was strongly reduced in the presence of D-limonene. Isolates that had previously shown strong or weak adherence, lost this capacity or had this capacity decreased (Figure 2A1,A2,B1,B2).

### 2.4. Evaluation of the D-Limonene Mechanism of Action

#### 2.4.1. Cell Death Profile

**Figure 3:**
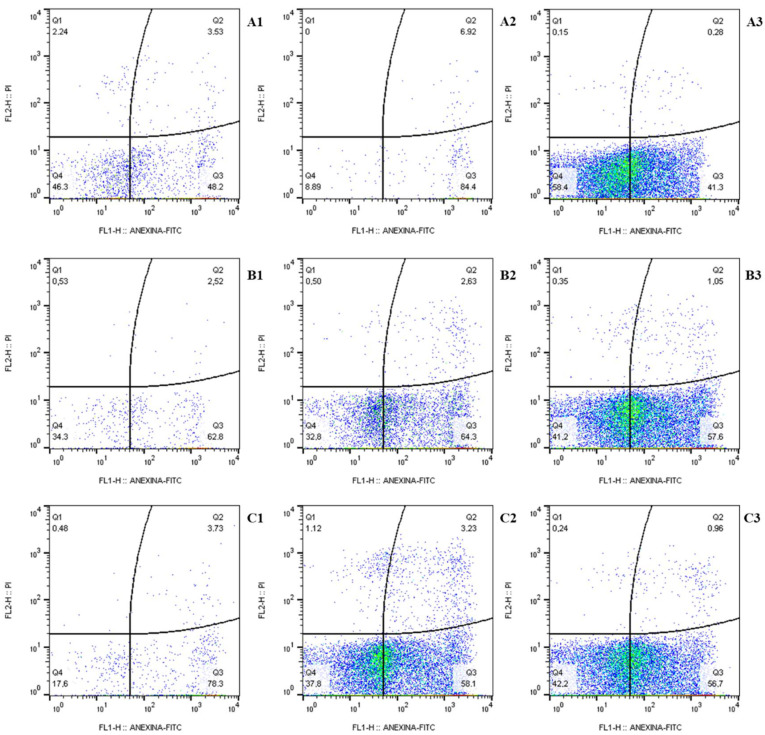
Flow cytometry for apoptosis analysis with Annexin V and propidium iodide in: (**A1**–**A3**) *Candida parapsilosis* stricto sensu URM7445 cells; (**B1**–**B3**) *C. orthopsilosis* URM 7434; (**C1**–**C3**) *C. metapsilosis* URM7423, 24 h post-D-limonene treatment. (**A1**–**A3**), cells exposed to 1024 µg/mL D-limonene, 512 µg/mL, and 256 µg/mL, respectively; (**B1**–**B3**), cells exposed to 512 µg/mL D-limonene, 256 µg/mL, and 128 µg/mL, respectively; (**C1**–**C3**), cells exposed to D-limonene 2048 µg/mL, 1024 µg/mL, and 512 µg/mL, respectively.

D-limonene treatment shows an initial apoptotic action (AV^+^/PI^−^ phenotype) that ranged from 41.3 to 84.4% for the *C. parapsilosis* URM7445 stricto sensu isolate (Figure 3A1–A3), from 57.6 to 64.3% for *C. orthopsilosis* URM7434 (Figure 3B1–B3), and from 56.7 to 78.3% for *C. metapsilosis* URM7423 (Figure 3C1–C3), marked by Annexin V (AV) in the analyzed cells. Moreover, the necrotic phenotype (AV^−^/PI^+^) was represented by less than 2.3% of the yeast population analyzed.

#### 2.4.2. Quantification of Reactive Oxygen Species (ROS) and Lipid Peroxidation

**Figure 4:**
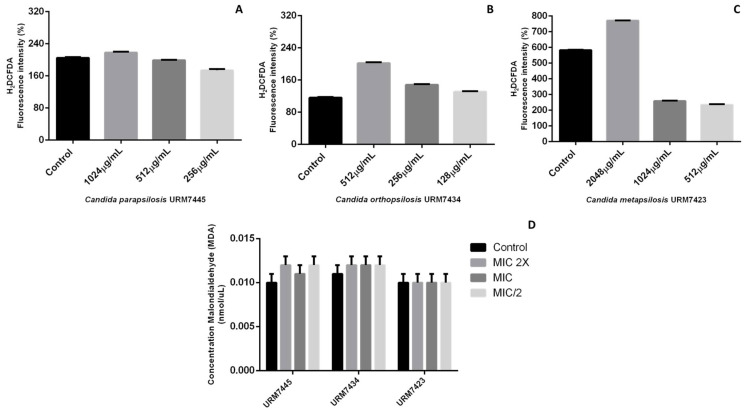
Detection of reactive oxygen species (**A**–**C**) and lipid peroxidation (**D**) for *Candida parapsilosis* complex species. For the analysis, a Tukey′s multiple comparison test was performed for all means obtained at a significance level of 5%.

The detection test for reactive oxygen species and lipid peroxidation is shown in Figure 4. The data pointed to greater ROS production at the concentrations of 2048 μg/mL and 512 μg/mL for the *C. metapsilosis* URM7423 and *C. orthopsilosis* URM7434 strains, respectively, while the *C. parapsilosis* URM7445 strictu sensu strain presented a slight increase in ROS production at 1024 μg/mL (Figure 4A–C). However, these data do not show statistical significance (*p* ≥ 0.5) as compared with the control.

#### 2.4.3. Mitochondrial Membrane Potential Evaluation

D-limonene did not cause mitochondrial membrane depolarization in the evaluated yeast strains. The URM7423 isolate showed variation rates of 0.44, 0.61, and 0.81 for the 2048 μg/mL, 1024 μg/mL, and 512 μg/mL concentrations, respectively. The same phenomenon occurred with the URM7445 isolates, with variation rates of 0.37, 0.39, and 0.41, and the URM7434 isolates, with variation rates of 0.63, 0.66, and 1.46 (respectively).

## 3. Discussion

This study revealed that D-limonene has antifungal activity against yeasts of the *C. parapsilosis* complex, in addition to having excellent antivirulence activity by inhibiting the morphogenesis and adherence of yeasts to the human epithelium. The antifungal activity of limonene was confirmed, as in the studies by Muñoz et al. [14] who found that, at concentrations ≥500 µM, it could inhibit the growth of *C. albicans*, *C. krusei*, *C. glabrata,* and *C. parapsilosis* in vitro. MIC values similar to those found in this study were also obtained by Ahmed et al. [15], with MIC values of 300 μg/mL for inhibition of *C. albicans* isolates. Furthermore, this terpene is widely used in the food, beverage, and cosmetic industries and is classified as a low toxicity additive, as cited by Ravichandran et al. [16]. Additionally, high MIC values for echinocandins such as those reported here are often found for *Candida parapsilosis* complex yeasts [17,18]. *C. orthopsilosis* has been reported to be resistant to caspofungin, as has *C. metapsilosis* [19,20,21]. The growing rate of resistance of yeast isolates to available drugs is alarming and has also been reported by several authors such as Lamoth et al. [22], who associated this growing resistance in yeast infections with the acquired or intrinsic resistance of the species, and the limitation of drugs in the therapeutic options is worrisome.

The budding-hyphal transition of *Candida* yeasts is a fundamental step for the establishment of the infection, contributing to tissue invasion [23]. Studies have shown that changes in fungal cell morphology caused by essential oils may be associated with an interference of enzymes responsible for cell wall biosynthesis or maintenance by its chemical constituents affecting fungal growth and morphogenesis [24,25]. In the present study, D-limonene reduced the ability of *C. parapsilosis* complex isolates to form germ tubes and pseudohyphae, characterizing this compound as a promising antivirulence agent.

Several studies with other yeast species have also evaluated the inhibition of morphogenesis by limonene. Liu et al. [26] and Brennan et al. [27] reported that limonene-mediated cell wall and membrane damage inhibited the growth of *Saccharomyces cerevisiae,* and therefore, its transformation into hyphae. Similar results were obtained by Thakre et al. [6] who evaluated the effect of limonene on the morphogenesis of *C. albicans*. However, this is the first study to evaluate the inhibition of morphogenesis by D-limonene in *C. parapsilosis* complex yeasts.

Regarding the inhibition of the ability to adhere to epithelial cells, the results of this study demonstrated that limonene was able to inhibit or reduce this virulence factor in vitro, when cultivated in the presence of this natural product. These data reinforce that, in addition to inhibiting growth, D-limonene can directly interfere with the adhesion of *C. parapsilosis* to the epithelium. Therefore, D-limonene may be particularly useful in the prevention of candidiasis, since the adhesion process is necessary for the establishment of the disease [28]. In addition, the inhibition of this virulence factor contributes to the non-development of biofilms, since adhesion is the initial stage for their formation and development. Furthermore, biofilms are responsible for increasing resistance to host immune defenses as well as antifungal therapy [29].

Adhesion inhibition of *Candida* species has also been verified with other natural products such as *Phyllanthus emblica* Linn. [30] and *Eugenia uniflora* extracts [31], both against *C. albicans*. However, there are no other studies that have demonstrated the inhibition of D-limonene adherence in *C. parapsilosis* complex yeasts.

Studies on the mechanism of cell death caused by limonene are still incipient. Flow cytometric assessment of apoptosis and necrosis is usually performed by the combined use of Annexin V-FITC, which accesses phosphatidylserine exposed on the outer membrane during the early phase of apoptosis, and propidium iodide (PI), which identifies nuclear changes during the final stage stages of apoptosis or necrosis as a consequence of increased membrane permeability [32]. The present results showed that the incubation of *C. parapsilosis* complex yeast cells with D-limonene produced significant losses in cell viability, causing apoptotic characteristics in the yeast. Furthermore, the observed cell death profile represents an important target for the development of new antifungal drugs, since the necrotic pathway can activate proinflammatory mechanisms in the human host [33].

D-limonene did not cause oxidative stress in *C. parapsilosis* complex cells in this study, yet the increase in ROS can be attributed to the strictly aerobic metabolism of these strains [34]. Furthermore, the lipid peroxidation assay showed no increase in relative MDA content in treatments as compared with the controls (Figure 4D). This effect supports the notion that D-limonene did not disturb the redox state of *C. parapsilosis* complex cells, and therefore, did not cause lipid peroxidation, since lipid peroxidation is the main molecular target of oxidative damage arising from species reactive [35].

In this study, damage to the mitochondrial membrane was not observed in yeasts of the *C. parapsilosis* complex treated with D-limonene. This may have occurred due to a peculiar characteristic of the mitochondria of the complex species, this being an uncoupling of oxidative phosphorylation, presumably caused by the presence of an uncoupling protein (UCP) [36].

In addition, strains URM7445 and URM7434 showed dependence on the dose of anidulafungin, with MIC values of 4 µg/mL, while strain URM 7427 showed resistance to this drug with an MIC value of 8 µg/mL, which may have contributed to the non-depolarization of the mitochondrial membrane, since cell wall disturbances by this antifungal may have led to the development of a resistance mechanism that contributed to resistance to oxidative stress [33].

Therefore, this is the first study to evaluate the antivirulent activity of D-limonene against *C. parapsilosis* complex yeasts and the results characterize this compound as promising for the treatment of invasive candidiasis, thus, contributing to a better prognosis and patient survival, since adhesion to epithelial cells and morphogenesis are crucial for the establishment and aggravation (biofilm formation) of fungal infections.

## 4. Materials and Methods

### 4.1. Candida Strains and Culture Conditions

Forty clinical isolates from the *Candida parapsilosis* complex obtained from the URM Mycotheca Culture Collection (Coleção de Culturas Micoteca URM) and the Sylvio Campos Medical Mycology Laboratory (Laboratório de micologia Médica Sylvio Campos), both from the Federal University of Pernambuco (Universidade Federal de Pernambuco), were analyzed, these being: *C. parapsilosis* stricto sensu (URM 6338, URM 6365, URM 6387, URM 6404, URM 6405, URM 6406, URM 6407, URM 6409, URM 6410, URM 6411, URM 6412, URM 6905, URM 6939, URM 6944, URM 6948, URM 7087, URM 7421, URM 7425, URM 7426, URM 7428, URM 7429, URM 7430, URM 7431, URM 7432, URM 7433, URM 7443, URM 7444, URM 7445, URM 7446, URM 7448, URM 7449, URM 7450, HAM17, MM12199, and HAM 26), *C. orthopsilosis* (URM 7434 and URM 7447), and *C. metapsilosis* (URM 6408, URM 7423, and URM 7427). In addition, *C. parapsilosis* ATCC 22019, *C. metapsilosis* ATCC96143, and *C. orthopsilosis* ATCC96141 reference strains were used. All strains were grown on the surface of a Sabouraud dextrose agar culture medium and incubated at 37 °C for 24 h before the experiments.

### 4.2. Antifungal Activity

The standard antifungals amphotericin B, fluconazole, anidulafungin, caspofungin, and micafungin, as well as D-limonene (Sigma Aldrich, St. Louis, MO, USA) were used for antifungal susceptibility testing, following the protocol proposed by the M27 A3 document [37,38]. The *C. parapsilosis* ATCC 22019 isolate was employed as a control. The RPMI 1640 (Sigma-Aldrich, St. Louis, MO, USA) culture medium with L-glutamine and without sodium bicarbonate, pH 7.0 ± 0.1, was used with morpholine propane sulfonic acid (MOPS, 0.165 mol·L^−1^, Sigma-Aldrich). Different concentrations of the antifungals were prepared according to the document.

The yeasts were maintained in Sabouraud dextrose agar (SDA) media and incubated at 35 °C. Isolate suspensions were prepared in saline and their density was adjusted according to the MacFarland 0.5 scale, at 90% transmittance, using a spectrophotometer at 530 nm wavelength. The inoculum volume was adjusted to 5.0 mL with sterile saline, and then diluted in RPMI 1640 to a concentration of 2–5 × 10^3^ cells/mL.

For the sensitivity tests, 96-well flat microtiter plates (TPP, Trasadingen, Switzerland) were used. The inoculum was added to the wells with the drugs to be tested at concentrations ranging from 0.03 to 16 µg/mL for amphotericin B, anidulafungin, caspofungin, and micafungin, and from 0.125 to 64 µg/mL for fluconazole, and from 2 to 1024 µg/mL for D-limonene, followed by plate incubation at 37 °C for 24 and 48 h. The minimum inhibitory concentrations (MIC) were determined with inhibitions ≥50% or even up to 100%, relative to the control well and depending on the antifungal.

### 4.3. Virulence Characterization of Yeast Isolates from the *C. parapsilosis* Complex and D-Limonene Antivirulence Activity

#### 4.3.1. Morphogenesis Assay

The assay was performed following Chaves et al. [39] in the presence and absence of D-limonene, in which *C. parapsilosis* complex yeast cells were grown for 24 h in NGY medium (Difco Neopeptone 1 g/L, dextrose 4 g/L, Difco yeast extract 1 g/L) (30 °C and 100 rpm). The absorbance of the optical growth density was determined using a spectrophotometer with a 600 nm wavelength between 0.8 and 1.2, which corresponded to an approximate concentration of 2 × 10^8^ cells/mL. Yeast cells were standardized to 1 × 10^6^ cells/mL, which corresponded to 50 μL of the growth culture encompassed between 0.8 and 1.2 absorbance.

For germination tube induction, the total yeast suspension volume was inoculated into YPD broth (2 g dextrose, 2 g peptone, 1 g yeast extract, and 100 mL distilled water) and incubated under mechanical shaking at 37 °C at 100 rpm for a period of 1 h and 3 h incubation for posterior microscopic reading.

In samples taken following 1 h of incubation, 100 yeast cells were observed under an optical microscopy (400× magnification, CX21, Olympus) to determine the percentage of germination tube formation. For samples incubated for a period of 3 h, the yeast cells were also recorded in the previously described manner for the morphology index calculation. Spherical yeast cells with blastoconidia morphology were assigned the IM value = 1; cells with a diameter twice their length, IM = 2; cells with pseudohyphal appearance, IM = 3; and long parallel hyphae, IM = 4. The following formula was used to determine the morphology index:

(1) $$I.M\;=\frac{\left(n{}^{\circ}\;\mathrm{IM}1\times 1\right)+\left(n{}^{\circ}\;\mathrm{IM}2\times 2\right)+\left(n{}^{\circ}\mathrm{IM}3\times 3\right)+\left(n{}^{\circ}\;\mathrm{IM}4\times 4\right)}{100}$$

#### 4.3.2. Adherence Capacity Assay of Yeast Cells to Epithelial Cells

Adherence tests were performed in the presence and absence of D-limonene, based on the studies by Kearns et al. [40] and Bates et al. [41]. Epithelial cells were obtained from the oral cavity of clinically healthy and caries-free young donors. Phosphate buffered saline (PBS) buffer was used in all steps relating to the adherence tests. The obtained cell suspensions were kept in an ice bath to avoid cellular alterations.

*Candida* isolates were seeded in Sabouraud dextrose agar medium with 0.5% yeast extract and maintained at 30 °C for 72 h. Following this period, the yeast cells were suspended in 2 mL of PBS contained in test tubes, centrifuged three times at 1580 rpm for 10 min, and resuspended to a final concentration of 2 × 10^7^ cells/mL.

Epithelial cell removal was performed by gentle scarification of the oral cavity mucosa with the aid of a swab, followed by suspension in 7 mL of PBS in test tubes, centrifugation three times over, and resuspension to a final concentration of 4 × 10^4^ cells/mL. After washing, yeast cells and epithelial cells were examined for viability and integrity to assess possible changes from the washes.

Mixing and homogenization was performed after obtaining *Candida* culture suspensions and oral cavity epithelial cells. Then, these were stirred for two hours and microscopy was performed following their preparation on a slide with methylene blue. The results were expressed by the arithmetic mean of ten observed fields, where 100 epithelial cells were evaluated with respect to the percentage of their surface area that was adhered to yeast cells and graded with a strong adhesion for adhesions covering 50% to 100% of the surface area, or poor adhesion for adhesions up to 49% or no visible adhesions.

### 4.4. Evaluation of the D-Limonene Mechanism of Action

One representative from each *C. parapsilosis* complex species (*C. metapsilosis* URM7423, *C. orthopsilosis* URM7434, and *C. parapsilosis* URM7445 sensu stricto) was used for the evaluation of the D-limonene mechanism of action.

#### 4.4.1. Cell Death Profile

Treated and untreated yeast cells were washed and resuspended in 0.5 mL of PBS and labeled with propidium iodide (PI) and Annexin V, using the Annexin V-FITC apoptosis detection kit (Ebioscience, San Diego, CA, USA), according to the manufacturer′s instructions. Then, the cells were immediately analyzed on a FACS-Calibur flow cytometer (Becton-Dickinson, San Jose, CA, USA) by means of a 530/30 nm signal detector (FL1-H) for AV-FITC detection and 585/42 nm signal detector (FL2) for PI emission. Fluorescence intensity was acquired for 20,000 events in the closed region. Data were analyzed using FlowJo (Tree Star Inc., Ashland, DE, USA) and expressed as the percentage of cells in each population phenotype as compared to the control (untreated cells). Two independent experiments were performed (*n* = 4) [32].

#### 4.4.2. Reactive Oxygen Species (ROS) Quantification

The 2’,7’-dichlorofluorescein (DCFH-DA) marker was used for reactive oxygen species (ROS) quantification. After the minimum inhibitory concentrations were determined in the antifungal sensitivity tests [20], samples were collected, washed with PBS, and labeled with 10 µL of the marker at a concentration of 10 µg/mL. Untreated D-limonene controls with and without DCFH-DA labeling were employed. The samples were analyzed by flow cytometry (FACS-Calibur/Becton-Dickinson, San Jose, CA, USA) and fluorescence intensity was acquired for 20,000 events in the closed region. Data were analyzed using FlowJo (Tree Star Inc., Ashland, DE, USA). Changes in DCFH-DA (FL1-H/515–545 nm) fluorescence intensities were quantified using the index of variation (IV) obtained by the equation (TM-CM)/CM, where TM is the median fluorescence for treated cells and CM is the median fluorescence for the control (untreated). Two independent experiments were performed (*n* = 4) [32].

#### 4.4.3. Malondialdehyde Assay (MDA)

The lipid peroxidation assay was determined by the MDA colorimetric reaction (532 nm) with thiobarbituric acid (TBA), using the MDA Assay Kit (Sigma-Aldrich, St. Louis, MO, USA) and following the manufacturer′s conditions.

#### 4.4.4. Mitochondrial Membrane Potential Evaluation

Treated and untreated cells were washed and resuspended in 0.5 mL of PBS with 10 mg/mL Rhodamine 123 (Sigma-Aldrich) for 20 min. After labeling, cells were washed twice with 1 mL of PBS and immediately analyzed by flow cytometry (FACS-Calibur/Becton-Dickinson, San Jose, CA, USA), where fluorescence intensities for Rhodamine 123 (mitochondrial membrane potential) were quantified. Fluorescence intensity was acquired for 20,000 events in the closed region. Data were analyzed via FlowJo (Tree Star Inc., Ashland, DE, USA). Changes in Rhodamine 123 (FL1-H/515–545 nm) fluorescence intensities were quantified using the index of variation (IV) obtained by the equation (TM-CM)/CM, where TM is the fluorescence median for treated cells and CM is the fluorescence median for the control (untreated). Two independent experiments were performed (*n* = 4) [32].

## 5. Conclusions

This study revealed that D-limonene exhibits antifungal activity against yeasts from the *C. parapsilosis* complex, in addition to presenting excellent antivirulence activity for inhibiting yeast morphogenesis and adherence to human epithelium. The fact that this compound affects cell walls and membranes, as reported by Thakre et al. [6], contributes to changes in the constituents of these structures, preventing adhesion to the epithelium and altering cell morphogenesis, leading to cell death. Furthermore, the apoptotic mechanism triggered by this compound represents an important target for the development of new antifungal drugs since it does not activate host inflammatory responses [37].

Of note, D-limonene did not cause oxidative stress in yeasts from the *C. parapsilosis* complex, as evidenced by the MDA assay; thus, apoptosis induction may be related to disorders in specific cell differentiation programs mediated by amino acids in the cell membrane triggered by the action of limonene [34].

This is the first study to evaluate D-limonene antivirulence activity against yeasts from the *C. parapsilosis* complex and the results characterize this compound as promising for the treatment of invasive candidiasis, hence, contributing to a better prognosis and patient survival, since adherence to epithelial cells and morphogenesis are crucial for the establishment and aggravation (biofilm formation) of fungal infections.

## Figures and Tables

**Table 1:** Antifungal susceptibility profile of *Candida parapsilosis* complex strains against commercial antifungals and D-Limonene.

Strains	AmB (µg/mL)	FLU (µg/mL)	CFG (µg/mL)	AFG (µg/mL)	MFG (µg/mL)	D-Limonene (µg/mL)
URM6338	1	1	1	4	2	512
URM6365	0.25	0.25	0.125	8	1	1024
URM6387	1	0.25	1	4	2	1024
URM 6404	1	0.5	0.25	2	0.125	256
URM6405	0.125	0.25	0.5	2	0.125	512
URM6406	0.5	0.125	2	2	2	512
URM6407	1	0.5	1	4	1	1024
URM6408	0.06	0.25	1	2	0.25	256
URM6409	0.5	0.125	2	2	2	512
URM6410	1	0.25	2	4	1	1024
URM6411	0.5	1	0.5	2	0.25	256
URM6412	0.125	0.125	4	4	2	>1024
URM6905	1	0.5	0.25	84	4	512
URM6939	1	0.5	2	4	0.06	>1024
URM6944	0.06	0.25	0.5	2	0.5	512
URM6948	0.25	1	4	4	0.5	>1024
URM 7087	0.5	0.5	0.25	2	0.125	256
URM 7421	1	0.25	0.25	8	4	512
URM7423	0.5	0.25	2	8	4	>1024
URM7425	0.125	0.25	2	8	2	1024
URM 7426	0.5	0.5	1	8	4	1024
URM 7427	0.125	1	0.125	4	1	512
URM7428	1	0.25	2	8	4	1024
URM 7429	0.25	0.25	0.25	4	2	1024
URM 7430	0.5	0.25	1	8	2	256
URM 7431	0.5	0.25	0.25	2	0.25	1024
URM7432	1	0.06	2	4	1	256
URM 7433	0.25	0.25	0.125	8	1	1024
URM 7434	0.125	1	0.125	4	1	512
URM7443	0.25	0.5	4	4	0.5	512
URM7444	0.06	0.125	0.25	2	0.5	>1024
URM 7445	0.5	1	0.125	4	1	256
URM 7446	0.5	1	1	8	4	1024
URM7447	1	0.06	2	4	1	512
URM7448	0.25	4	4	8	2	256
URM7449	0.5	0.5	2	8	2	1024
URM7450	0.25	0.06	2	4	0.5	>1024
HAM17	0.125	0.5	4	4	0.5	1024
MM12199	1	0.25	1	8	2	>1024
HAM26	0.125	0.25	2	8	0.5	512

Legend: AmB—amphotericin B; FLU—fluconazole; CFG—caspofungin; AFG—anidulafungin; MFG—micafungin.

## Data Availability

Not applicable.

## Sample Availability

Samples of the compounds are available from the authors.
